# Less-invasive fascia-preserving surgery for abdominal wall desmoid

**DOI:** 10.1038/s41598-021-98775-2

**Published:** 2021-09-29

**Authors:** Yoshihiro Nishida, Shunsuke Hamada, Tomohisa Sakai, Kan Ito, Kunihiro Ikuta, Hiroshi Urakawa, Hiroshi Koike, Shiro Imagama

**Affiliations:** 1 Department of Rehabilitation Medicine, Nagoya University Hospital, 65 Tsurumai, Showa, Nagoya, Aichi 466-8550 Japan; 2 Department of Orthopaedic Surgery, Aichi Cancer Center Hospital, 1-1 Kanokoden, Chikusa-ku, Nagoya, 464-8681 Japan; 3 Department of Orthopaedic Surgery, Nagoya University Hospital, 65-Tsurumai, Showa-ku, Nagoya, 466-8550 Japan

**Keywords:** Medical research, Oncology

## Abstract

The mainstay of treatment for desmoid has been shifted to active surveillance (AS). However, surgery is still being performed on abdominal wall desmoid with a wide surgical margin. The purposes of this study are to clarify the treatment results of less-invasive, fascia preserving surgery for patients with abdominal wall desmoid, and to propose a new treatment modality. Since 2009, 34 patients with abdominal desmoid have been treated in our institution. Among them, as a final treatment modality, 15 (44%) were successful with AS, 15 were subjected to less-invasive surgery, and 4 methotrexate and vinblastine treatment. The clinical results of less-invasive surgery were clarified. In the surgical group, although the surgical margin was all microscopic positive (R1), only one patient (6.7%), who has the S45F mutation type of CTNNB1, showed recurrence, at a mean follow-up of 45 months. There were no patients with familial adenomatous polyposis (FAP)-related desmoid in this cohort. Only two patients (13%) required fascia lata patch reconstruction after removal of the tumor. In patients with non FAP-related abdominal wall desmoid, less-invasive, fascia preserving surgery is recommended as a favorable option as active treatment. Based on the results of this study, multi-institutional further research is warranted with an increased number of patients.

## Introduction

Desmoid-type fibromatosis (desmoid) is a proliferative disease of (myo)fibroblast-like cells that is classified as an intermediate tumor according to the World Health Organization classification. It tends to infiltrate surrounding tissues, but does not metastasize^1,2^.

The recurrence rate, after high quality surgery aiming for a negative surgical margin, was reported in the range of 20 to 60% in the past reports from overseas^3–5^, and the recurrence rate has been reported to be similar in Japanese^6,7^. The recurrence rate has also been found to be higher in children and adolescents^8^.

For these reasons, the treatment modality for desmoid has changed in recent years, and the policy is to first follow up with active surveillance (AS) without performing surgery^9,10^. However, when the AS policy fails, it is necessary to consider active treatment. Regarding surgical treatment, postoperative results differ greatly depending on the site of occurrence, and it has been reported that the recurrence rate is particularly low for abdominal wall desmoids^11–13^. A consensus paper based on a review of these pieces of evidence has provided a treatment algorithm including surgical options after AS for abdominal wall desmoid, which is different from those in other locations^9^.

Regarding the relationship between surgical margin and postoperative recurrence for desmoid, an increasing number of research reports have indicated that there is no association between resection margins (R0 vs R1) and recurrence rates^3,5,7,11,13,14^. If there is no difference in the results between the method of removing only the macroscopic tumor and microscopic negative margin surgery for abdominal wall desmoid, non-invasive surgery has advantages for patients.

However, in the previous reports of surgery for abdominal wall desmoid aiming at a negative surgical margin, there were disadvantages to patients, such as abdominal wall defects caused by surgery and the need for mesh reconstruction^15,16^ or plastic surgery reconstruction^17^.

We have reported favorable results of surgery even with microscopic positive margin for patients with truncal desmoid including six patients with abdominal wall desmoid^18^. However, this report did not include patients with results based on the AS policy, or detailed surgical data (surgery time, bleeding volume, postoperative reconstruction).

The purpose of this study is to evaluate the treatment results of the less-invasive, fascia-preserving surgery that we have prospectively performed for patients with abdominal wall desmoid. In particular, we analyzed the recurrence rate after surgery with R1 surgical margin, the necessity of reconstruction, and the involvement of CTNNB1 mutation. In addition, we would like to clarify the significance of this surgical procedure in the treatment strategy for abdominal wall desmoid where AS becomes the mainstream.

## Materials and methods

**Figure 1:**
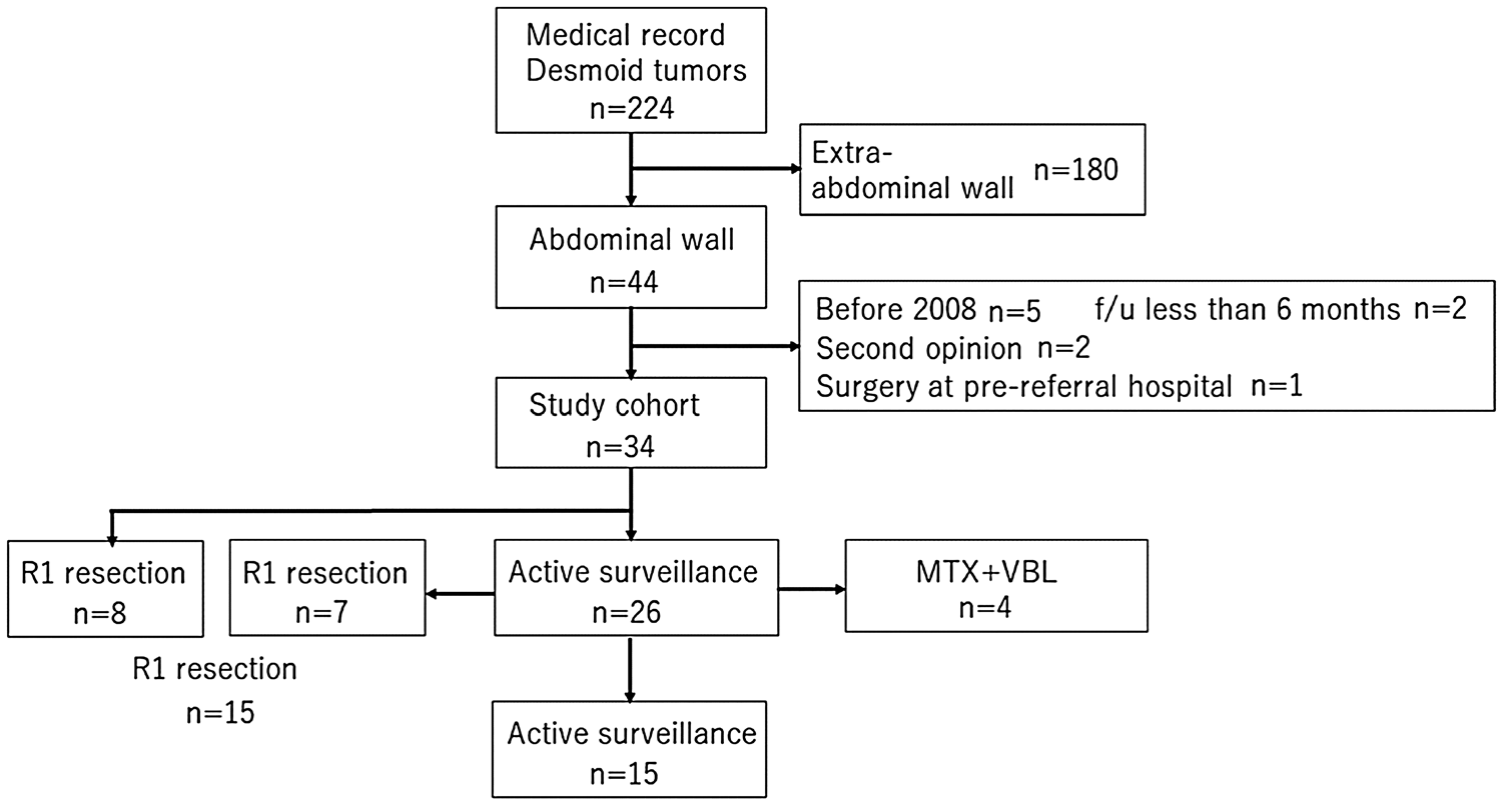
Flowchart of the present study. Flowchart shows the inclusion of patients in the present study. (Adobe photoshop CS6 ver.13.0 × 32, Microsoft PowerPoiont 2013).

According to the medical records, between June 1991 and October 2020, 224 patients were diagnosed with desmoid and treated at our institution, of which 44 (19.6%) developed on the abdominal wall. Since 2003, the treatment modality for abdominal wall desmoid at our institution recommends non-surgical treatment initially with administration of the selective COX-2 inhibitor meloxicam^19,20^, but since July 2017, AS without NSAIDs including COX-2 inhibitor has been employed as the initial treatment policy, which is in accordance with the present consensus guideline^9^. For patients with growing tumors of the abdominal wall and/or impaired ADL/QOL such as severe pain, surgical treatment with R1 resection has been recommended to patients^18^ since January 2009. For patients who refuse surgical treatment, we recommend MTX and VBL treatment as another option with evidence of efficacy^21–23^. Except for abdominal wall development, if the tumor size increases according to the AS policy, and the ADL/QOL disorder worsens, surgery is not recommended, but MTX + VBL or pazopanib treatment is recommended. Of 44 patients with abdominal wall desmoid, after excluding 5 before 2009 when R1 resection was started, 2 who visited our outpatient clinic for a second opinion, 2 with follow-up of less than 6 months, and one who underwent surgery at another hospital, 34 patients were included in this study (Fig. 1). Four patients refused surgical treatment when the tumor size increased, and so received MTX + VBL treatment.

According to the consensus paper on desmoid treatment, no report has documented a high level of evidence regarding the effect of NSAID treatment^9^. Therefore, patients treated with meloxicam were included in the AS group in the present study. Abdominal wall desmoid was defined as development from the rectus abdominis muscle, oblique muscle, or transverse abdominal muscle.

AS at our institution is evaluated by MRI or CT once every three months. If the condition settles down, this is done once every 6 months.

Indications for surgery were decided in consultation with patients by explaining the outline of surgery, predicted recurrence rate, length of hospital stay etc. when the tumor size increases or symptoms such as pain worsen.

A hotspot mutation in the β-catenin gene, CTNNB1, is known to be the cause of the onset of desmoid, with the results of surgical treatment differing depending on this mutation type^6,24–26^. At our institution, once the pathological diagnosis of desmoid is made, the mutation type of CTNNB1 is analyzed by the Sanger method in all patients^19^.

**Figure 2:**
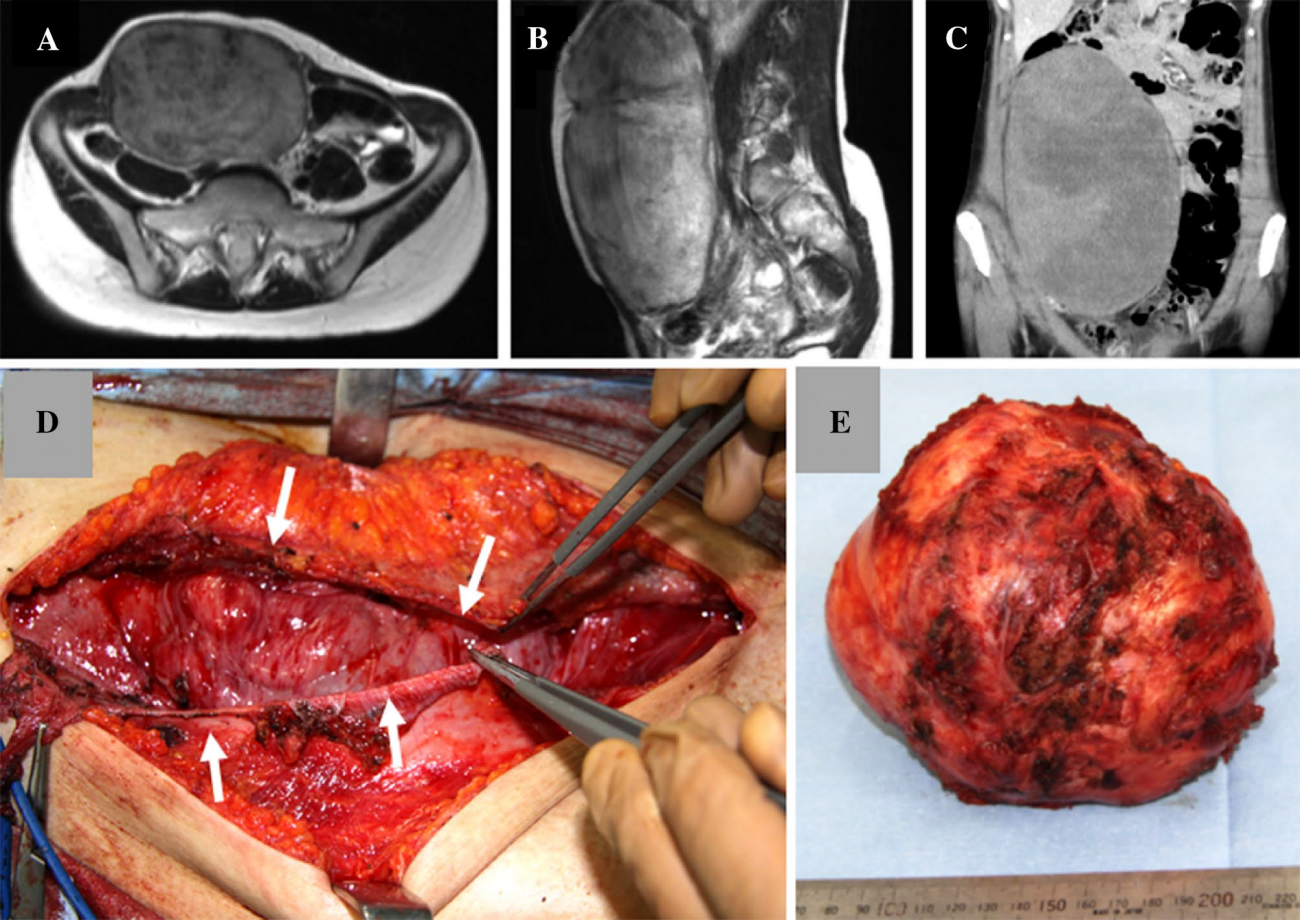
Preoperative images, and findings during fascia-sparing surgery. Patient 1 (**A**–**D**). Preoperative MRI and CT. T2-weighted axial plane (**A**), T2-weighted sagittal plane (**B**), Contrast-enhanced sagittal plane of CT (**C**). Fascia (white arrows) was preserved after removal of tumor (**D**). Patient 4. Removed desmoid detached from fascia (**E**). (Adobe photoshop CS6 ver.13.0 × 32, Microsoft PowerPoiont 2013).

Our surgical procedure for abdominal wall desmoid is different from surgery with a marginal margin. Unlike the excision in the reaction layer on the margin of the tumor, the tumor is macroscopically exposed, and detached from the fascia and muscle, and the fascia is preserved as much as possible. This makes it occasionally difficult to control bleeding from the tumor surface. On the other hand, since the fascial defect is minimal after tumor resection, wounds can be generally closed without reconstruction, such as mesh (Fig. 2).

We analyzed various clinical factors, CTNNB1 mutation status and oncological outcome in the group of less-invasive surgery. We also compared the differences in various factors between the surgery group, AS group, and MTX + VBL treatment group. Radiological response of AS or MTX + VBL treatment was evaluated according to Response Evaluation Criteria in Solid Tumors (RECIST, version 1.1)^27^. In the case of surgery, if there was no recurrence, it was evaluated as CR, and if it recurred, it was evaluated as stable disease (SD), partial remission (PR), or progressive disease (PD) according to the change in size. This study was approved by the ethics committee of our institution (registration number: 2014–0217), and undertaken under the provisions of the Declaration of Helsinki. All of the participating patients signed informed consent forms.

### Statistical analysis

Comparisons between groups were performed on categorical variables using the chi-square test or Fisher's exact test. The Shapiro–Wilk test was performed to test the normality of the data. The Wilcoxon signed rank test was used for nonparametric analysis between the two corresponding groups. The t-test or one-way analysis of variance was used for the comparison of means in parametric data between two or more groups. Kruskal–Wallis test was used for comparison of median in nonparametric data between the three groups. LRFS was estimated using the Kaplan–Meier method. All statistical analysis was performed using SPSS version 20. P < 0.05 was considered statistically significant.

### Institutional review board statement

This study was approved by Institutional Review Board of Nagoya University Hospital.

### Informed consent statement

Informed consent was obtained from all subjects involved in the study.

## Results

Of the 34 patients, 31 were women, the mean age at the first visit was 36 years (range: 19–68), and the median maximum tumor size at the first visit was 74 mm (range: 31–223). Patients with familial adenomatous polyposis (FAP)-related desmoid were not included.

The initial treatment modality was AS in 26 patients, and surgery was performed in 8 patients. In these 8 patients, AS was performed at other universities or specialized facilities for bone and soft tissue sarcoma. When the tumor size increased significantly and/or the activities of daily living (ADL)/quality of life (QOL) disorder became worse, they were referred to our hospital. Therefore, there were 8 patients who underwent surgery at our institution without a period of AS. This seems to be the cause of the selection bias between the surgery group and the AS group in our institution.

Of the 26 patients with AS, eleven had tumor growth or worsening symptoms. Tumor growth was evaluated as PD by RECIST without setting an observation period. As a criterion for changing to active treatment, it was not decided by the evaluation of pain by the numerical rating scale. Instead, when the patient complained of ADL impairment due to pain, changes to active treatment were considered. Among them, seven patients underwent surgery and 4 patients were subjected to methotrexate (MTX) + vinblastine (VBL) treatment. Therefore, finally, surgical treatment was performed in 15 patients (44%), only AS policy in 15 patients (44%), and MTX + VBL in 4 patients (12%) (Fig. 1).

Of the 15 patients with surgical treatment, 14 were female, 12 patients (80%) had pain at the first visit, the mean age at the time of surgery was 36 years (range: 20–52), and the median maximum tumor diameter at the time of surgery was 11.6 cm (range: 4.5–22.3). The CTNNB1 mutation types were T41A in 6 patients, T41I in 3 patients, S45F in 1 patient, H36P in 1 patient, p.Ser45_Gly48del in 1 patient, and wild type (WT) in 3 patients. The median period from the first visit to our hospital to surgery was 3 months, the median operation time was 125 min (range: 53–338), and the median bleeding volume was 116 ml (range: 10–2762). Only two patients had a defect in the abdominal fascia after tumor resection, and the other 13 patients did not require reconstruction because the fascia could be preserved. In two patients with a defect, the abdominal wall was repaired simply by applying a fascia lata patch with a diameter of about 5 × 5 and 15 × 10 cm, respectively. No mesh reconstruction was required. Pathologically, all patients had a microscopic positive margin (R1 resection).

The mean and median postoperative follow-up period for patients with surgery was 45 and 38 months, respectively. Only one patient (6.7%) developed a recurrence 16 months after surgery, and interestingly it was the only patient harboring S45F mutation. In this patient, the tumor size was stable after recurrence, and then spontaneously regressed. Five-year local recurrence-free survival (LRFS) rate was 92.3%, and estimated LRFS time was 80.6 months ± 5.2 months (confidence interval 70.5–90.8). Detailed information of patients with surgical treatment is provided in Table 1. No patients had any serious postoperative complications including hernia, and preoperative pain disappeared. No patients complained of postoperative pain because they did not require the use of mesh for reconstruction, and no ADL / QOL issues were noted.

**Table 1:** Patients with abdominal wall desmoid treated with tumor excision.

Age	Gender	Size	Pain	CTNNB1	Surg time	Bleeding	Margin	Recon	F/U	Rec
30	F	18.0	+	del	154	112	R1	‒	86	‒
20	F	13.0	+	S45F	338	500	R1	+*	120	+
39	F	8.4	‒	T41A	104	58	R1	‒	85	‒
36	F	14.0	+	T41A	195	622	R1	‒	66	‒
40	F	12.0	+	WT	125	378	R1	‒	38	‒
36	F	4.5	+	T41A	53	43	R1	‒	74	‒
43	F	6.6	‒	T41A	60	52	R1	‒	41	‒
26	M	6.5	+	T41I	82	37	R1	‒	24	‒
33	F	10.2	+	H36P	108	116	R1	‒	41	‒
35	F	5.8	+	T41A	58	10	R1	‒	24	‒
38	F	10.8	+	WT	96	448	R1	‒	24	‒
52	F	18.0	‒	T41I	153	726	R1	‒	19	‒
39	F	22.3	+	WT	308	2762	R1	+*	16	‒
40	F	11.6	+	T41I	143	90	R1	‒	6	‒
36	F	13.6	+	T41A	150	636	R1	‒	6	‒

*Age* at surgery, *Size* maximum diameter of tumor (cm), *CTNNB1* mutation type, *Surg time* surgery time (minutes), *Bleeding* intraoperative bleeding volume, *Margin* microscopic surgical margin, *Recon* plastic reconstruction after tumor excision, *F/U* follow up duration (months), *Rec* recurrence, *F* female, *M* male, *del* deletion, p.Ser45_Gly48del with whole exome sequencing, *Patch with fascia lata.

Of the 15 patients whose progress was monitored only by AS, 7 were evaluated as complete remission (CR) (Table 2). Table 3 shows a comparison of the 15 patients with AS, 15 with fascia-preserving surgery, and 4 with MTX + VBL treatment (comparison between 3 groups). The tumor size was significantly different (p = 0.038). This was the salient difference between the surgery (11.6 cm) and AS (6.2 cm) groups in multiple comparisons using Tukey’s test. There were significantly more disease-free patients in the surgery group regarding the final oncological status (p = 0.025).

**Table 2:** Patients with treatment of only active surveillance.

Age	Gender	Size 1	Size 2	Pain	CTNNB1	Treatment	F/U	RECIST
36	F	6.9	0	+	WT	+	117	CR
35	F	6.2	0	+	WT	+	46	CR
36	F	13.5	0	+	T41A	+	80	CR
65	M	8.1	8.5	+	T41A	+	39	SD
38	F	6.5	7.7	‒	T41A	+	20	SD
33	F	5.8	4.2	+	S45F	+	10	PR
35	F	6.0	0	‒	NA	+	41	CR
68	M	5.5	0	‒	WT	+	43	CR
35	F	3.6	0	+	T41I	‒	39	CR
37	F	3.1	0	+	NA	+	31	CR
37	F	9.3	9.5	+	T41A	+	25	SD
22	F	8.5	8.7	+	T41A	‒	20	SD
30	F	4.2	4.0	+	T41A	‒	9	SD
39	F	7.5	7.2	+	WT	‒	12	SD
30	F	6.1	6.1	‒	T41A	‒	6	SD

*Age* age at first visit to our hospital, *Size 1* maximum tumor diameter at first visit (cm), *Size 2* maximum tumor diameter at last visit (cm), *Treatment* meloxicam or celecoxib treatment at pre-referral or our hospital, *F/U* follow up duration (months), *RECIST* evaluation between first and last visit, *NA* not available due to the poor quality of DNA from desmoid (pre-referral hospital), *CR* complete remission, *PR* partial remission, *SD* stable disease.

**Table 3:** Comparison between active surveillance only, surgery, and MTX + VBL treatment group.

	Active surveillance	Surgery	MTX + VBL	P value
Number of patients	15	15	4	
Age at first visit	36	36	30	0.21
Gender (male)	2	1	0	0.94
Size at first visit	6.2	11.6	9.1	0.038
Pain+	11	12	2	0.40
CTNNB1				0.95
T41A	7	6	0	
T41I	1	3	0	
S45F	1	1	1	
Others	0	2	1	
WT	4	3	2	
NA	2*	0	0	
F/U duration	31	40	45	0.49
Status at last visit				0.025
Disease free, CR	7	14	0	
With disease, PR + SD	8	1	4	
With disease, PD	0	0	0	

*Age, size, F/U* median value, *MTX* methotrexate, *VBL* vinblastine, *WT* wild type, *F/U* median follow up, duration from first visit to last visit, *CR* complete remission, *PR* partial remission, *SD* stable disease, *PD* progressive disease.

*2 cases were excluded due to the low quality of DNA.

Next, we focused on and analyzed the 26 patients who selected AS for the initial treatment strategy (Suppl. Table 1). Fifteen patients (58%) were able to continue AS, 7 patients (27%) switched to surgery and 4 patients (15%) switched to MTX + VBL treatment because of tumor growth and/or worsening pain. The median age was 36 years for AS, 32 years for surgery, and 29.5 years for MTX + VBL, which were lower than in the group selected for active treatment, but not significantly different (p = 0.184). The median tumor size at the first visit was AS 6.2 cm, surgery 7.3 cm, and MTX + VBL 9.1 cm (p = 0.13). In the patients switched to surgery, the tumors had increased in size significantly at the time of surgery (10.2 cm) compared to that at the first visit (7.3 cm) (p = 0.028, Wilcoxon signed rank test). In the MTX + VBL group, the tumor tended to grow from the first visit (9.1 cm) to the start of treatment (11.1 cm) (p = 0.068).

## Discussion

In desmoid including abdominal development, it is recommended to follow the course with AS as much as possible^9,10^. On the other hand, a systematic review of studies analyzing AS revealed that the median reported percentage of shifting to an active treatment was 29% during the course of AS. As for active treatment, it is reported that systemic treatment was the most common, followed by surgery^28^. In the present study, of the 34 patients studied, all patients were treated with initial AS in pre-referral hospital or our institution. Fifteen of 34 (44%) was successful with AS. After failure of AS, the present study indicated that less invasive surgery is a good option for abdominal wall desmoid.

The present study revealed that the recurrence rate of abdominal wall desmoid is very low (6.7%) even with less-invasive, fascia-preserving surgery. Table 4 summarizes 6 past reports^15,16,29–32^ and the present study on the surgical results of abdominal wall desmoid. The recurrence rate noted in each of these reports was very good, from 0 to 16%, and the total recurrence rate of the past 6 reports was 6.2% (9/145). Bonvalot et al. reported that 18 of 41 patients had a surgical margin of R1, despite which only 1 patient showed recurrence^29^. Of the 145 patients, R0 or wide resection was performed in 96 (66%) with a recurrence rate of 6.2%. It is very interesting that the recurrence rate (6.7%) of the present study, which was all R1 surgery, was equivalent, suggesting the importance of less-invasive surgery compared to surgery with a wide surgical margin. According to a report from a multicenter joint study from Japan, only 1 of 13 patients (7.7%) with abdominal wall involvement recurred including both R0 and R1 margin, which is also equivalent to the results of the present study^6^.

**Table 4:** Studies reporting results of abdominal wall desmoid with surgery.

Author	Patients no.	Age (median)	Male	Tumor size (median ) (cm)	CTNNB1	Surgical margin	Recurrence
Sutton	6*	28.5 (mean)	0 (0%)	11.7	NA	Wide: 6**	1 (16%)
Bertani	14	35	3 (21%)	4.7 (mean)	NA	R0: 13, R1:1	0 (0%)
Catania	7	35 (mean)	1 (14%)	NA	NA	R0: 7	0 (0%)
Bonvalot	41	34	3%***	5	NA	R0: 23, R1: 18	1 (2.4%)
Wilkinson	50	36	2 (4%)	8	NA	R0: 22, R1: 28	4 (8%)
Couto Netto	27	34	3 (11%)	10	NA	R0: 25, R1: 2	3 (11%)
This study	15	36	1 (7%)	11.6	T41A:6 T41I:3 S45F:1 Others: 2 WT: 3	R1: 15	1 (6.7%)

*One case with FAP-related desmoid excluded.

**Information for microscopic margin not provided.

***Actual number is unclear. All cohort (147 cases) ratio.

*NA* not available.

Although the postoperative results of abdominal wall desmoid are good, several factors need further consideration. The first is the clinical question of what the surgical margin of abdominal wall desmoid should be. As shown in Table 4, the overall postoperative results are extremely good despite the total of 64 patients of R1 included in the past 6 reports. Combined with the results of the present study, it is suggested that less-invasive, fascia preserving (R1) surgery is acceptable for desmoid arising in the abdominal wall.

Second, as related to the surgical margin, it would be beneficial for patients if reconstruction could be avoided after tumor resection. Sutton et al., Bertani E et al., and Cataniar et al., performed immediate plastic reconstruction (mesh) in all patients after tumor excision^15,30,32^. In Bonvalot’s study, 27 (66%) of 41 patients required full-thickness abdominal wall mesh repair. For 17 patients who underwent surgery after AS, mesh was used in all^29^. In our surgical procedure, only two patients required a fascia lata patch, with all of the others avoiding reconstruction.

Third is the clinical question of whether the CTNNB1 variant affects surgical outcomes. In a recent meta-analysis summarizing seven studies, the authors concluded that S45F is a risk factor for local recurrence after surgery compared to T41A, S45P, and WT^33^. On the other hand, no studies focusing on abdominal wall desmoid have been reported. The previous reports shown in Table 4 do not include CTNNB1 data either. However, it is noteworthy that the sole recurrent patient in the present study harbored S45F mutation type.

The fourth is the clinical question of whether background of abdominal wall desmoid (sporadic or FAP-related) affects surgical outcomes. A previous study analyzing the results of surgical treatment for FAP-related desmoid reported that, of 12 abdominal wall desmoids, 8 were completely resected macroscopically, and recurred in 4 patients^34^. This suggests that even with abdominal wall desmoid, the recurrence rate is expected to increase when FAP-related.

Regarding whether the rate of changing to active treatment differs depending on the site of occurrence, Turner et al. found no differences in the risk of progression during AS between abdominal wall tumor and other sites^35^. Another study demonstrated that the 5-year progression free survival of primary cases managed with AS of trunk/thoracic wall tumors and abdominal wall tumors was similar^10^. These results mean that the treatment modality for abdominal desmoid needs to be changed from AS to active treatment at a certain rate.

From the results of the present study, unlike other sites, we recommend less-invasive, fascia preserving surgery rather than systemic treatment as an active treatment for the abdominal wall desmoid. In addition, as shown in Table 3, the high rate of oncological status becoming disease free in the surgery group may be of psychological benefit to patients compared with those in the AS and MTX + VBL groups.

There are several limitations in the present study. The AS cohort included patients treated with meloxicam. However, this is consistent with the systematic review of AS that similarly included studies using non-steroidal anti-inflammatory drugs (NSAIDs). There was no evidence that NSAIDs were effective against desmoid^9^. Although the recurrence rate of abdominal wall desmoid is low, it is still unclear whether CTNNB1 status, especially S45F, is implicated in its recurrence. In the present study, only 15 patients were analyzed, and it is necessary to accumulate more patients in multiple centers to determine whether the recurrence rate is really low with less-invasive and fascia preserving surgery.

## Conclusions

For abdominal wall desmoid, less invasive surgery that preserves the fascia has a low recurrence rate and generally does not require reconstruction, despite having an R1 margin. In abdominal wall desmoid, unlike other sites, less invasive surgery might be recommended over systemic treatment when active treatment is required after AS. Further research with an increased number of patients is warranted to verify the significance of this procedure.

## Supplementary Information

Supplementary Information.

## Data Availability

The research data is available in a data base repository in our institution, and can be available upon reasonable request.
