# Supplementary material for: Less-invasive fascia-preserving surgery for abdominal wall desmoid
Source: Sci Rep. 2021 Sep 29;11:19379. doi: 10.1038/s41598-021-98775-2 (PMC8481551; doi:10.1038/s41598-021-98775-2)
Supplement: Supplementary file 1 — Supplementary Information. [file 41598_2021_98775_MOESM1_ESM.docx]

Table S1. Twenty-six cases with initial active surveillance.

|  | Initial active surveillance (n=26) | | |  |
| --- | --- | --- | --- | --- |
|  | Active surveillance | Surgery | MTX+VBL | P value |
| Number of patients | 15 | 7 | 4 |  |
| Age at first visit | 36 | 32 | 30 | 0.184 |
| Gender (Male) | 2 | 1 | 0 | 0.92 |
| Size at first visit | 6.2 | 7.3 | 9.1 | 0.13 |
| Size at treatment | N.A. | 10.2 (P=0.028*) | 11.1 (P=0.068*) | 0.65** |
| Pain + | 11 | 6 | 2 | 0.82 |
| CTNNB1  T41A  T41I  S45F  Others  WT  NA | 7  1  1  0  4  2 | 3  1  1  2  0  0 | 0  0  1  1  2  0 | 0.95 |
| Status at last visit  disease free, CR  with disease, PR+SD  with disease, PD | 7  8  0 | 6  1  0 | 0  4  0 | 0.22 |

Age, size: median value, MTX: methotrexate, VBL: vinblastine, WT: wild type, CR: complete remission, PR: partial remission, SD: stable disease, PD: progressive disease

*compared to size at first visit (Wilcoxon signed rank test)

** compared of size at treatment between surgery and MTX+VBL
